# Adult pancreatoblastoma with atypical histological morphology combined with familial adenomatous polyposis: a rare case report

**DOI:** 10.3389/fonc.2024.1346964

**Published:** 2024-02-28

**Authors:** Ying-Xia Wang, Su-Su Fan, Xue-Rong Peng, Yu-Shan Zhu, Xuan Zhang

**Affiliations:** ^1^ Department of Pathology, the First Affiliated Hospital of Kunming Medical University, Kunming, China; ^2^ Yunnan College of Modern Biomedical Industry/School of Pharmaceutical Sciences & Yunnan Key Laboratory of Pharmacology for Natural Products, Kunming Medical University, Kunming, China

**Keywords:** pancreatoblastoma, familial adenomatous polyposis, atypical histological morphology, epithelial islands, differential diagnosis

## Abstract

Pancreatoblastoma (PB) is a rare malignant pancreatic epithelial tumor that mostly occurs in children and occasionally occurs in adults. The tumor has acinar cell differentiation and squamous corpuscles/squamous epithelial islands, which are frequently separated by fibrous bundles. Familial adenomatous polyposis (FAP) is an autosomal dominant inherited disease characterized by the presence of numerous adenomatous polyps in the colon and rectum. Cases of pancreatoblastoma combined with familial adenomatous polyposis (FAP) are rarely reported. A review of a rare case of adult pancreatoblastoma with atypical histological morphology combined with familial adenomatous polyposis is presented herein. In this case, the patient was first diagnosed with familial adenomatous polyposis and subsequently found to have pancreatoblastoma 1 year and 3 months later. This suggests pancreatoblastoma may occur in patients with familial adenomatous polyposis or a family history of the condition, indicating a possible association between the two tumors. Therefore, pancreatoblastoma should be included in a differential diagnosis for FAP patients with a pancreatic mass. The final diagnosis of pancreatoblastoma depends on the pathological diagnosis. Acinar-like cells and squamous corpuscles/squamous epithelial cell islands under light microscopy are the key diagnostic points. This case report also can improve the awareness of clinicians, radiologists, and pathologists on the presence of rare tumor-adult pancreatoblastoma in patients with familial adenomatous polyposis.

## 1 Introduction

Pancreatoblastoma (PB) is a rare malignant neoplasm that originates from pancreatic epithelial cells (1). With an incidence of approximately 0.004 per 100,000 cases every year, PB is one of the most common pancreatic tumors (2, 3). Pancreatoblastoma mostly occurs in children, often under 10 years old, with an average age of 4 years old, and it is rare in adults (4, 5).

Pancreatoblastoma often presents with nonspecific clinical symptoms and is often incidentally discovered as a solitary nodule in the abdomen, which can spread extensively. The pathological histological features include acinar cell differentiation and the presence of squamous corpuscles/squamous epithelial islands, along with varying degrees of endocrine and ductal differentiation. In the second-generation sequencing of adult pancreatoblastoma, APC mutations were found to be the most common mutations, indicating that APC mutation is the primary driver of this tumor (6). The APC/β-catenin signaling pathway has also been identified in 67% of pancreatoblastomas, with biallelic inactivation of the APC gene and activating mutations of the CTNNB1 (β-catenin) gene (7).

Familial adenomatous polyposis (FAP) is an autosomal dominant inherited disease characterized by the presence of numerous adenomatous polyps in the colon and rectum and is associated with a near 100% lifetime risk of colorectal cancer (CRC) (8). FAP is caused by germline mutations in the adenomatous polyposis coli (APC) gene, which is located on the long arm of chromosome 5 (5q21-22) (9). The global prevalence of FAP is ~1/10,000 in individuals, with men and women being affected equally (10). In addition to the gastrointestinal manifestations, FAP patients may exhibit extraintestinal manifestations, including gastric fundic gland polyps, fibromas, osteomas, epidermoid cysts, and pituitary adenoma (11, 12).

Both pancreatoblastoma and FAP are associated with APC mutations, suggesting that pancreatoblastoma may be a tumor related to FAP. However, it remains uncertain whether FAP independently increases the risk of developing pancreatoblastoma. To date, cases of pancreatoblastoma combined with FAP are rarely reported. In this study, we present a rare case of adult pancreatoblastoma with atypical histological morphology combined with familial adenomatous polyposis.

## 2 Case report

This study presents a rare case of adult pancreatoblastoma with atypical histological morphology combined with familial adenomatous polyposis in a 28-year-old male patient.

### 2.1 Clinical diagnosis and treatment process

**Figure 1:**
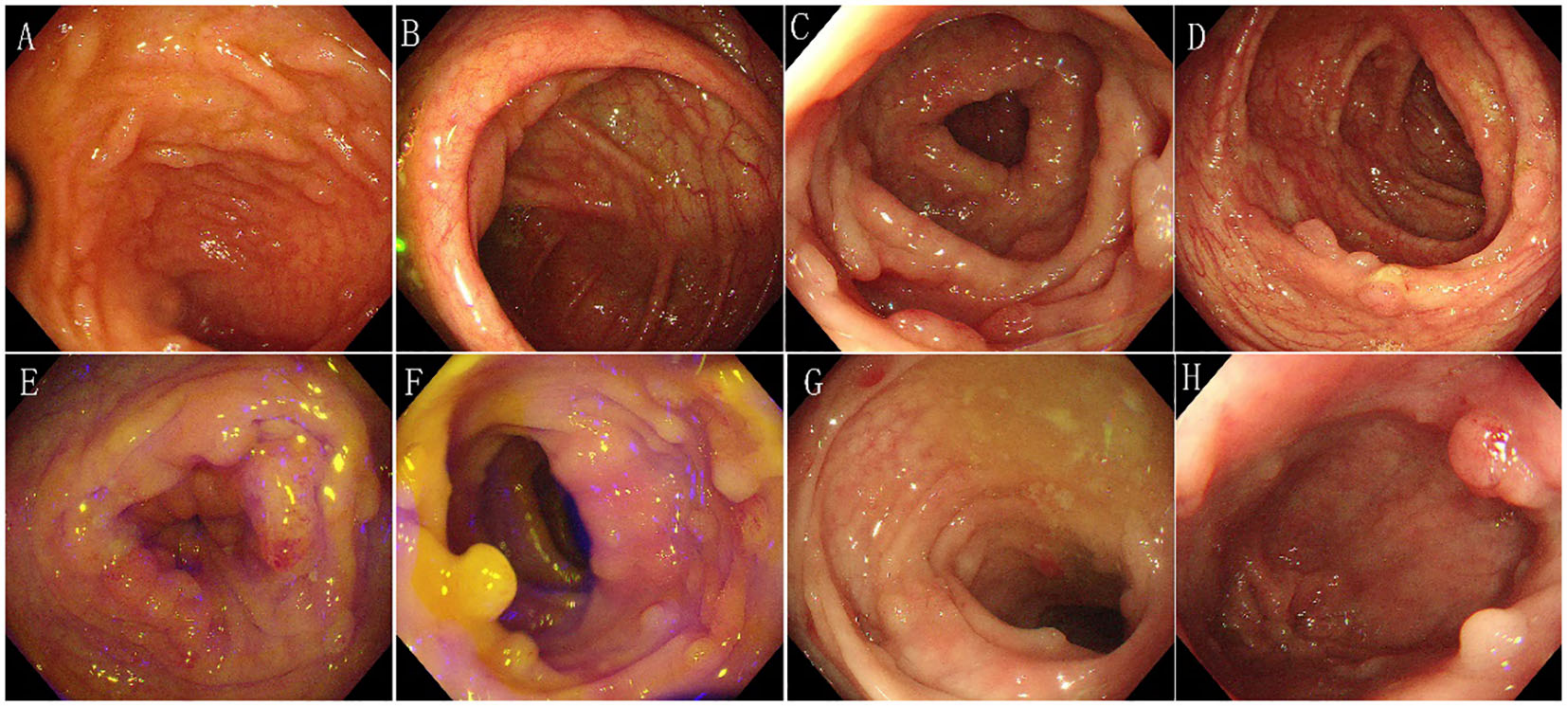
Characteristics of electronic colonoscopy. Over 100 polyps of varying sizes ranging from 0.3cm to 1.1cm in diameter were observed in the entire colon and rectum. **(A) **Terminal ileum - no abnormalities were observed. **(B)** Ileocecum - several polyps of varying sizes were observed. **(C)** Ascending colon - several polyps of varying sizes were observed. **(D)** Transverse colon - several polyps of varying sizes were observed. **(E)** Descending colon - several polyps of varying sizes were observed. **(F)** Sigmoid colon - several polyps of varying sizes were observed. **(G)** Rectum-sigmoid junction - several polyps of varying sizes were observed. **(H)** Rectum - several polyps of varying sizes were observed.

In February 2017, the patient visited the Department of Gastroenterology at the First Affiliated Hospital of Kunming Medical University due to recurrent loose stools, active bowel sounds in the morning and evening, occasional abdominal pain (relieved after passing gas), and mucus in the stool. An electronic colonoscopy examination revealed the presence of dozens of polyps ranging in size from 0.4cm to 1.2cm in diameter in the ileocecal region, as well as throughout the entire colon and rectum. Subsequently, the patient underwent polypectomy three times in February, May, and December 2017, with 11-13 polyps removed during each procedure. The pathological diagnosis after polypectomy was “tubular adenoma”. Following nutritional support therapy, the patient was discharged. In May 2018, the patient presented with abdominal cramps of unknown cause. An abdominal X-ray examination revealed partial intestinal gas accumulation and multiple small gas-liquid planes in the epigastrium. The patient was then diagnosed with incomplete intestinal obstruction and received analgesia and fluid replacement therapy. Another electronic colonoscopy examination revealed the presence of over 100 polyps ranging in size from 0.3cm to 1.1cm in diameter from the ileocecal region to the rectum (**Figure 1**), leading to a diagnosis of familial adenomatous polyposis(based on the family history: This patient’s grandfather, father and brother died of gastrointestinal malignant tumor).

**Figure 2:**
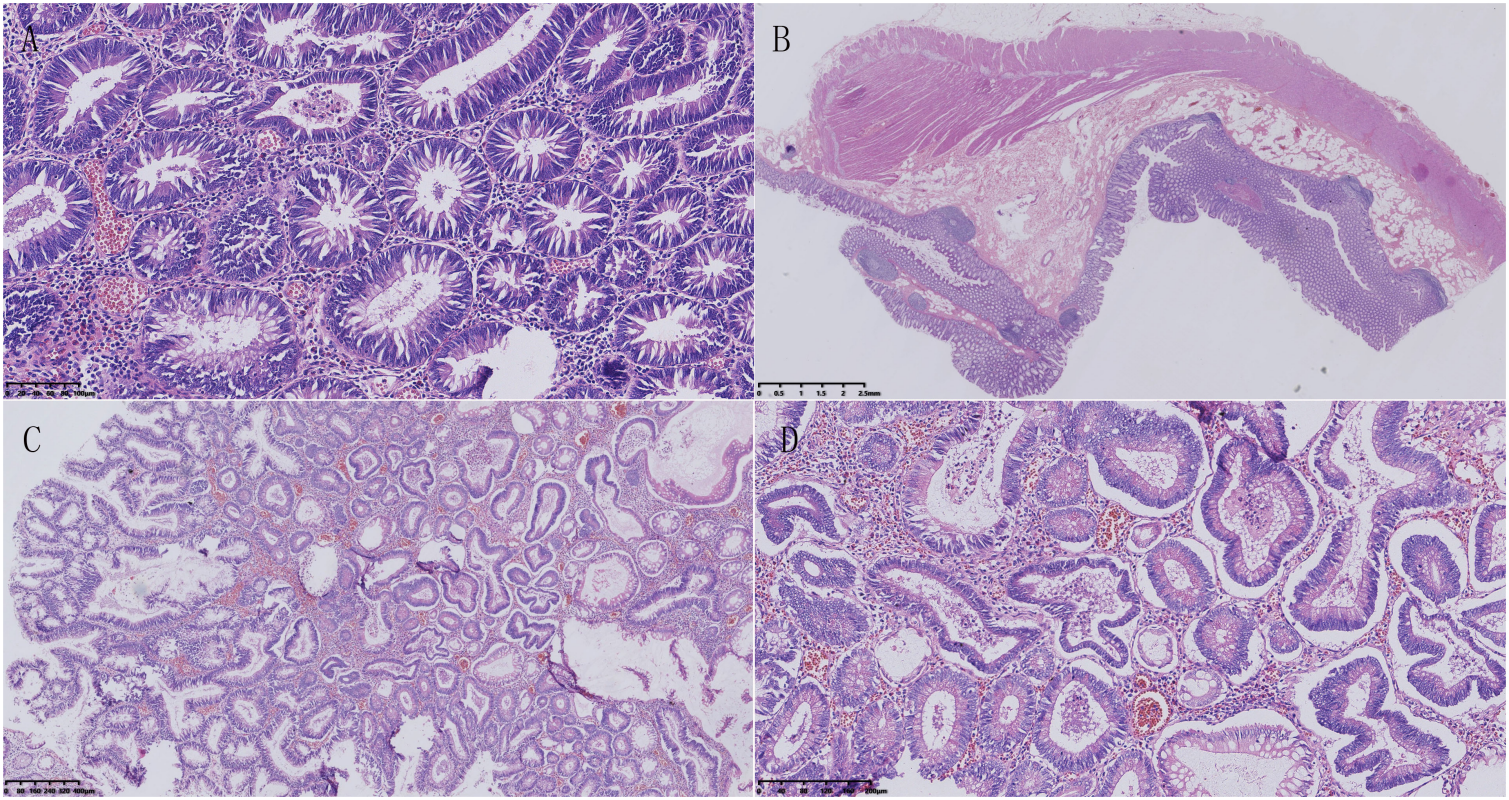
Pathological features of familial adenomatous polyposis. **(A)** Microscopic examination showed that the tumor consisted of tubular structures, with crypt cells in the colon proliferating and crowding together. The nuclei of the cells were elongated and deeply stained, showing pseudo-stratification. **(B)** Multiple polyps were visible in the colon, protruding above the mucosal layer, and extending into the intestinal lumen. No abnormalities were observed in the submucosal layer, muscular layer, or serosal layer. **(C)** The tumor cells were composed of two types of structures: villous and tubular. The villous structures were slender and finger-like, with a fibrovascular axis in the center. **(D)** Some glandular cells had enlarged and rounding nuclei, and significantly reduced cytoplasm. Most of the nuclei were located near the luminal surface of the cell, and few goblet cells were present, indicating characteristics of a high-grade intraepithelial neoplasm.

In addition, an abdominal CT scan revealed a small nodule in the pancreas, with a serum alpha-fetoprotein (AFP) level of 1.1ng/ml and other tumor markers within the normal range. In June 2018, the patient underwent total colorectal resection and resection of pancreatic body and tail mass. The pathological diagnosis was “multiple tubular-villous adenomas of the colon and rectum with focal high-grade intraepithelial neoplasia” (**Figure 2**). Additionally, there was a suspicion of pancreatic neuroendocrine carcinoma with high differentiation that needed to be differentiated from pancreatoblastoma. AE1/AE3, low molecular weight CK, CK7, β-catenin, MGMT, Villin, Synaptophysin, Chromogranin-A, and P53 were detected by immunohistochemical staining. However, APC mutation was not detected in this case. Regrettably, this case was also not discussed at a multidisciplinary tumor board, as it had not been established in the First Affiliated Hospital of Kunming Medical University until 2020. The doctors tended to think of it as pancreatic neuroendocrine carcinoma with high differentiation, and according to the Eighth Edition of the AJCC TNM Staging System of Pancreatic Neuroendocrine Tumors, the pathological stage was T2N0M0(the size of the tumor was 3.0×1.5×1.0 cm; no lymph node metastasis and distant metastasis). Therefore, the patient was not given chemotherapy after the first pancreatectomy.

**Figure 3:**
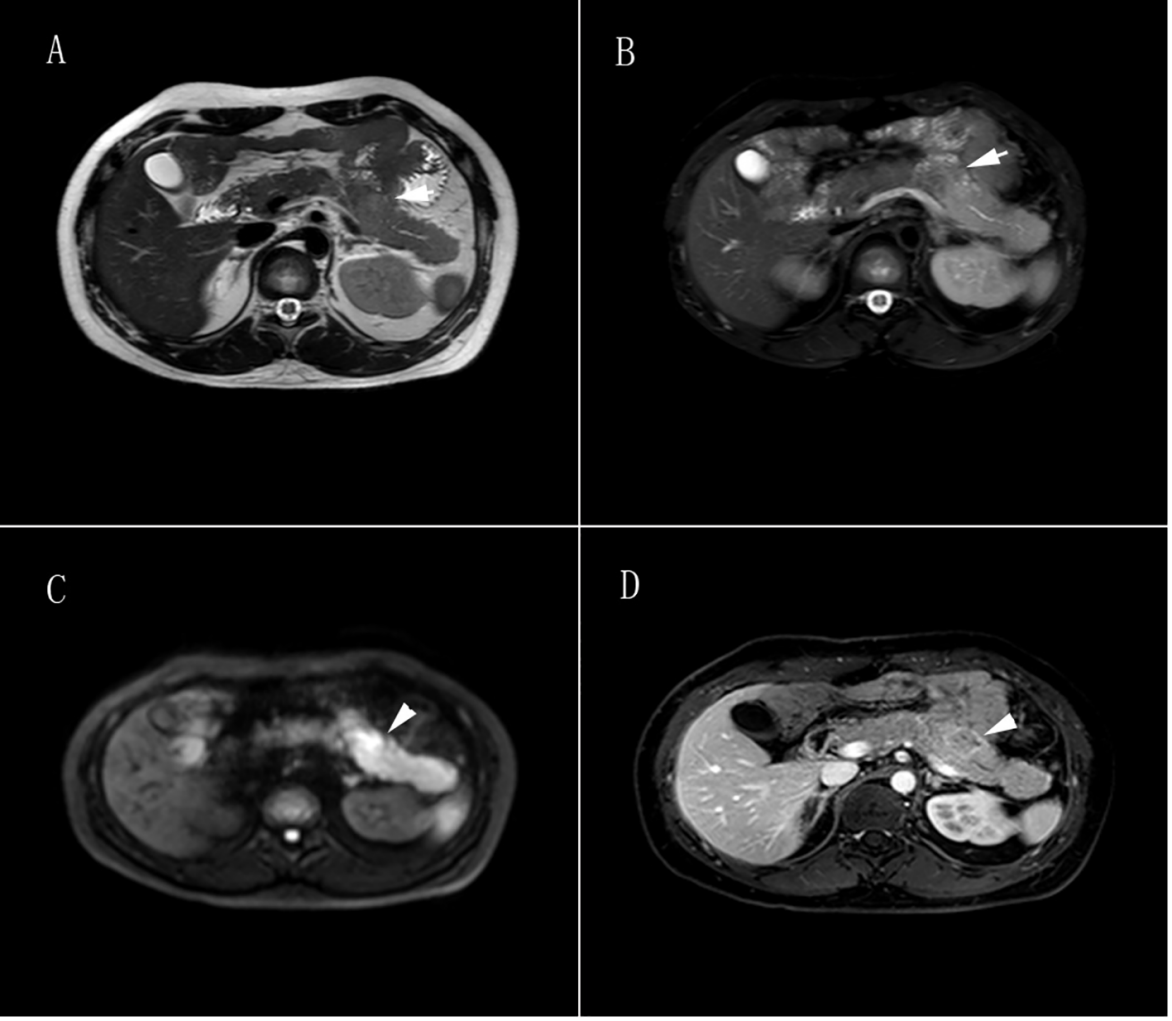
Pancreatic tumor MRI. **(A)** T1-weighted image showed irregular patchy and slightly enhanced signals in the body of the pancreas, with unclear borders and slight dilation of the main pancreatic duct nearby, slightly enhanced signals were found in the distal pancreatic tail of the lesion. **(B)** T2-weighted image showed irregular patchy and slightly enhanced signals in the body of the pancreas, with unclear borders and slight dilation of the main pancreatic duct nearby, slightly enhanced signals are found in the distal pancreatic tail of the lesion. **(C)** Diffusion-weighted imaging showed a high signal in the body of the pancreas. **(D)** Contrast-enhanced scan showed a progressive uneven enhancement signal in the body of the pancreas, with a ring enhancement signal as the main feature. The lesion measured 2.5cm × 2.7cm × 1.8cm (left-right × anterior-posterior × superior-inferior), and the distal pancreatic tail of the lesion showed a uniform enhancement signal.

**Figure 4:**
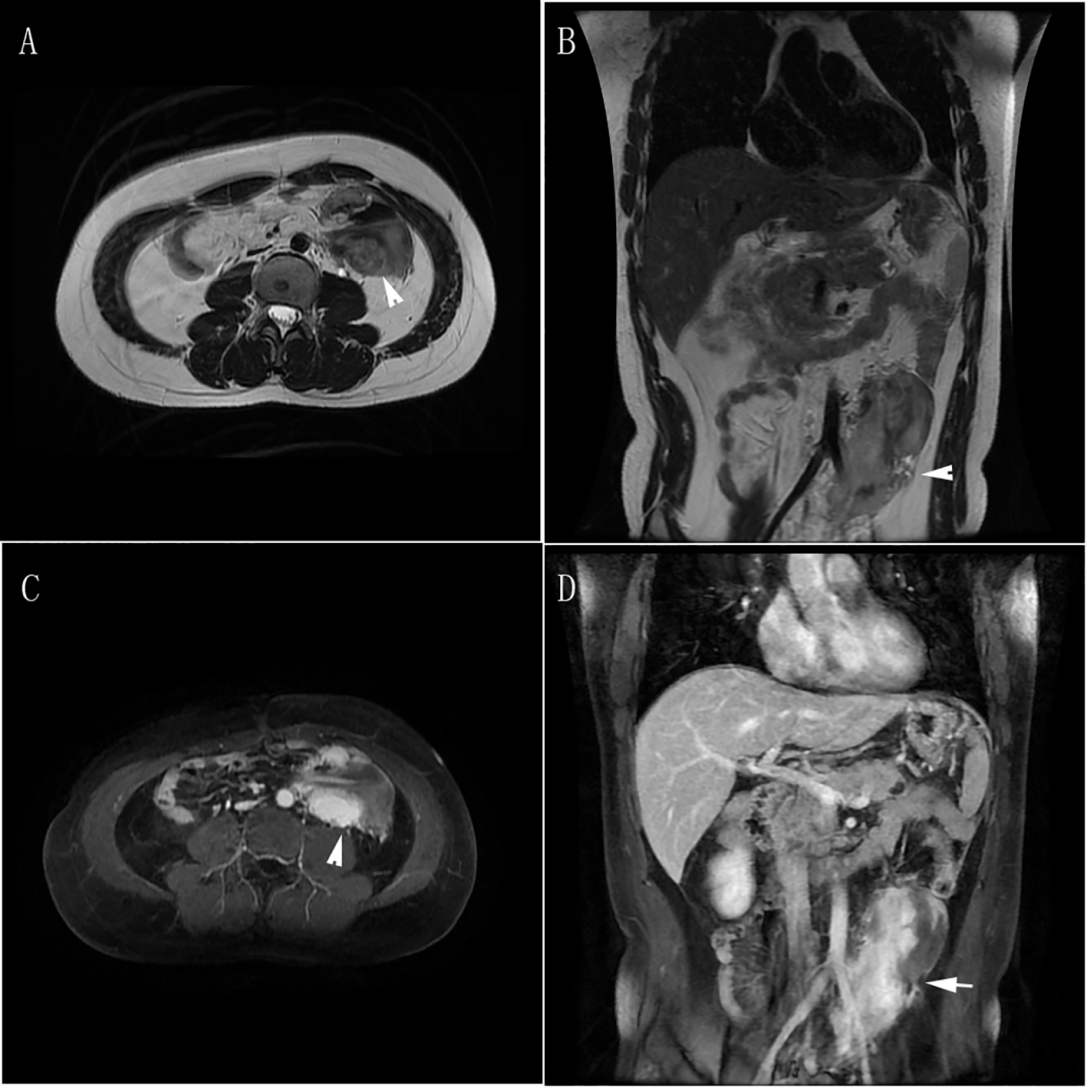
Abdominal metastases MRI. **(A, B)** T2-weighted images in transverse and coronal planes showed postoperative changes in the tail of the pancreas, with a mass-like/slightly lower T2 signal in the left middle abdomen. The boundary of the lesion was unclear, and it appeared to encircle part of the left iliac vessels and the middle segment of the ureter. The lesion measured 6.3cm × 5.8cm × 11.9cm, with suspected invasion of the psoas major muscle. Multiple small lymph nodes were visible in the abdomen. **(C, D)** Contrast-enhanced scans in transverse and coronal planes showed uneven enhancement signals of the lesion in the left middle abdomen.

Seven months after the initial surgery (January 2019), a space-occupying lesion was detected once again in the pancreatic tail. Following one month of observation (February 2019), an MRI revealed an increased pancreatic mass, indicating the recurrence of the tumor (**Figure 3**). The patient underwent another pancreatic tail resection, and the pathological analysis confirmed the same diagnosis as before, which was suspected pancreatic neuroendocrine carcinoma with high differentiation that needed to be differentiated from pancreatoblastoma. In order to obtain a more definitive diagnosis and establish a treatment plan, the pathological sections of the pancreatic tumor were sent to five highly regarded tertiary hospitals in China for consultation with renowned pathological experts. Ultimately, the final diagnosis was determined to be pancreatoblastoma (**Figure 4**).

Two months after the second pancreatic tail resection (April 2019), MRI revealed a mass in the left mid-lower abdomen. One month later (May 2019), a PET-CT revealed a mass in the same location, which involved the left ureter. The mass exhibited moderately active glucose metabolism and negative somatostatin receptor imaging. Additionally, the mass had significantly increased in size compared to April. The patient was diagnosed with tumor metastasis and pituitary adenoma. Subsequent MRI scans in June 2019 displayed a mass in the left middle abdomen measuring 6.3×5.8×11.9cm. This mass involved the left iliac vessels and the middle segment of the ureter. The boundaries were unclear, and there was suspicion of invasion into the Psoas major muscle. Furthermore, multiple small lymph nodes were visible in the abdominal cavity (**Figure 4**).

The patient underwent three cycles of chemotherapy at our hospital from August to September 2019. The chemotherapy regimen consisted of Vincristine 2mg on day 1, Cyclophosphamide 1.0g on day 2, Etoposide 0.1g from day 1 to day 3, and Cisplatin on day 3, day 4, and day 5. Following the third cycle of chemotherapy, an MR scan revealed an increase in the size of the mass in the left middle abdomen to 8.5×5.7×15cm, indicating a poor response to the chosen chemotherapy regimen. Meanwhile, the patient was not satisfied with the chosen chemotherapy regimen because of intolerance of adverse reactions such as gastrointestinal reactions. Despite recommendations for other chemotherapy regimens and further imaging examinations, the patient declined and requested to be discharged. Then the patient was discharged and received regular phone follow-ups.

**Figure 5:**
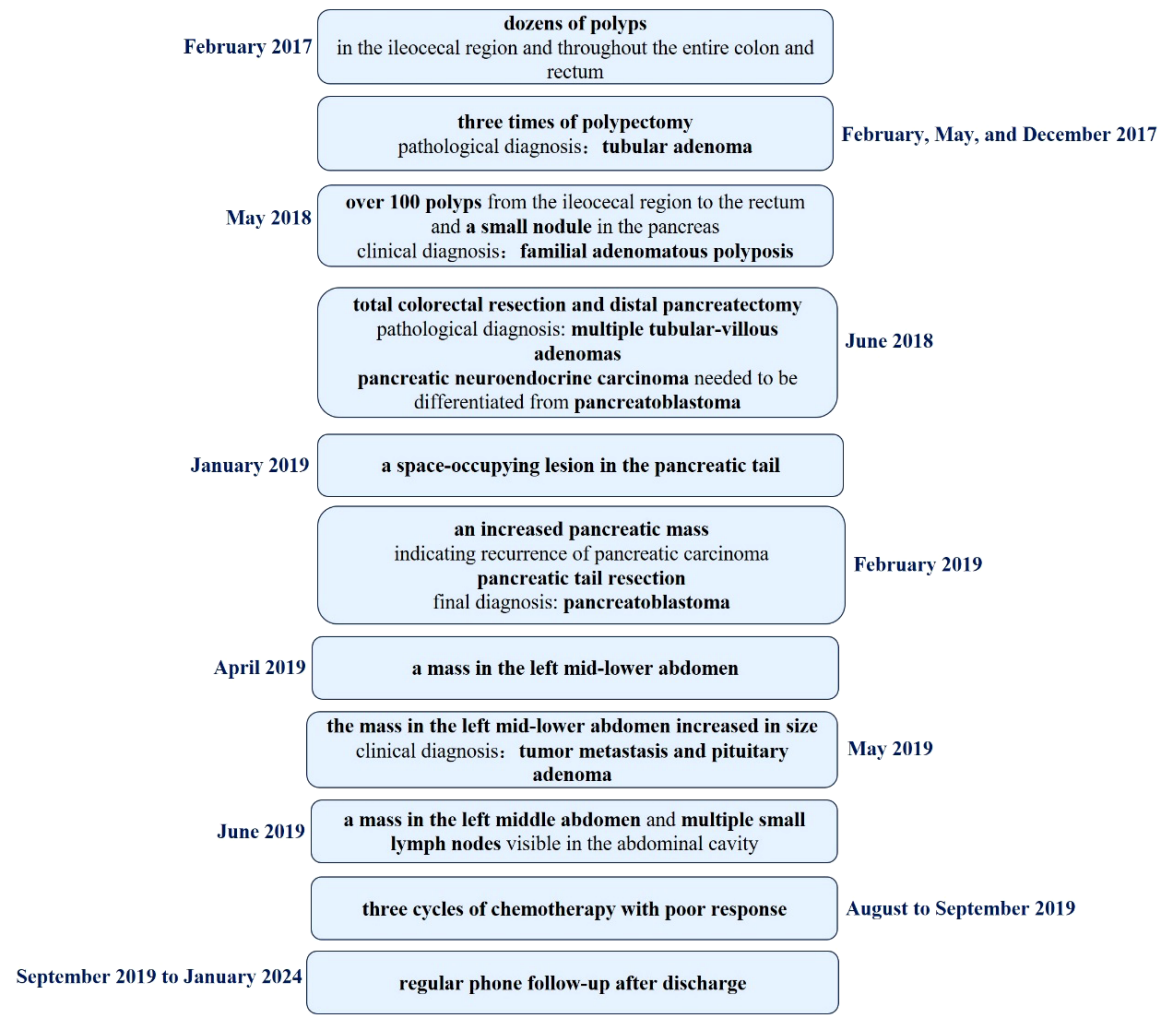
The schematic history of diagnosis and treatment of this patient.

The schematic history of diagnosis and treatment of this patient is summarized in **Figure 5**.

### 2.2 Pathological features of pancreatoblastoma

**Figure 6:**
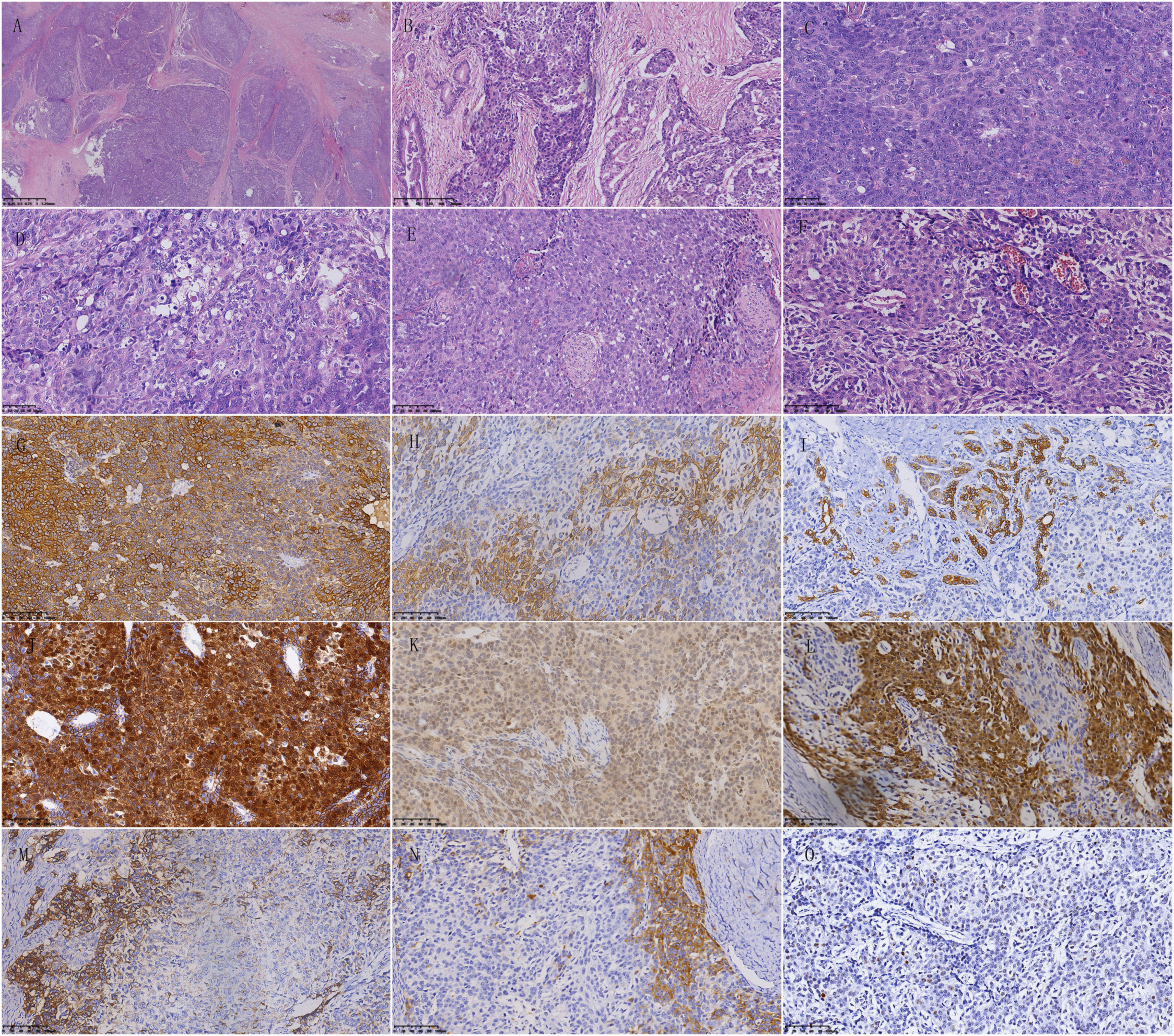
Pathological features of pancreatoblastoma. **(A)** The tumor cells are abundant and separated by fibrous septa, forming well-defined islands of epithelial cells. **(B)** Most tumor cells form solid nests, while a few cells form glandular structures. **(C)** Polygonal cells with acinar differentiation form solid nests around small cavities. The nucleoli are prominent, and the cells have moderate nuclear atypia. Mitotic figures are easily visible. **(D)** Some cells have abundant cytoplasm. **(E)** A few cells with abundant and pale cytoplasm are polygonal or spindle-shaped, forming islands of epithelial cells resembling squamous corpuscles. **(F)** Cells with neuroendocrine differentiation surround blood vessels. The chromatin is fine and dark and is evenly distributed in the nucleus, with an unclear nuclear membrane. **(G)** Cytoplasm positivity for AE1/AE3 in immunohistochemical staining. **(H)** Part of tumor cells show cytoplasm positivity for low molecular weight CK in immunohistochemical staining. **(I)** Part of tumor cells show cytoplasm positivity for CK7 in immunohistochemical staining. **(J)** Cytoplasm and nucleus positivity for β-catenin in immunohistochemical staining. **(K)** Cytoplasm positivity for MGMT in immunohistochemical staining. **(L)** Part of the tumor cells show cytoplasm and cell membrane positivity for Villin in immunohistochemical staining. **(M)** Neuroendocrine differentiated cells show cytoplasm positivity for Synaptophysin in immunohistochemical staining. **(N)** Neuroendocrine differentiated cells show cytoplasm positivity for Chromogranin-A in immunohistochemical staining. **(O)** Scattered few cells show nucleus positivity for P53 in immunohistochemical staining.

The pancreatic mass contained numerous tumor cells that were separated by fibrous septa into distinct epithelial cell islands. These islands were arranged in large nests and exhibited acinar cell differentiation and atypical squamous body-like structures. Some cells also showed neuroendocrine differentiation. The polygonal cells with acinar cell differentiation formed solid cell nests around small cavities, displaying obvious nucleoli and moderate nuclear atypia. The cells with neuroendocrine differentiation had thin and deep chromatin that was evenly distributed in the nucleus, with unclear nuclear membranes. Additionally, a few cells had abundant cytoplasm and formed epithelial cell islands resembling squamous corpuscles (**Figures 6A–F**).

Immunohistochemical staining results showed AE1/AE3 positive, Synaptophysin partially positive, Chromogranin-A partially positive, SSTR2 partially positive, CD56 partially positive, β-catenin nuclear positive, SMAD4 positive, CK19 partially positive, CK5/6-negative, P63-negative, AAT and AACT positive in scattered individual cells, MGMT positive, BCL-10-negative, CDX2 positive, Vllin positive, CEA-negative, Ki-67 positive in 10% cells, low molecular weight CK positive, VEGFR2 positive, and CK8/18 positive (**Figures 6G–O**).

### 2.3 Prognosis

The patient was diagnosed with pancreatoblastoma 1 year and 3 months after the onset of familial adenomatous polyposis. Seven months after the resection of the pancreatic tumor, the tumor recurred. Three months after the second resection of the pancreatic tumor, metastasis was found in the left middle abdomen, and the mass progressively increased in size, along with multiple enlarged lymph nodes in the abdominal cavity. The patient was unresponsive to the chosen chemotherapy regimen, and declined to receive other chemotherapy regimens. Then the patient was discharged and given regular phone follow-ups. Remarkably, the patient has survived for 4 years and 11 months since the diagnosis of pancreatoblastoma till January 2024(last follow-up). According to the patient’s description, the general condition remains good. However, the details of the treatment after discharge were unknown, and the imaging data were not obtained.

## 3 Discussion

Familial adenomatous polyposis(FAP) is an autosomal dominant syndrome characterized by the presence of numerous adenomatous polyps throughout the colon and rectum, which have a tendency to progress to adenocarcinoma (13). In this case, dozens of polyps were found throughout the colon and rectum, which were confirmed by pathology as tubular adenomas or tubular-villous adenomas. Some areas of these polyps also showed progression to high-grade intraepithelial neoplasia, including *in situ* carcinoma. It is important to note that some FAP patients may develop extraintestinal manifestations, such as thyroid and pancreatic cancer, hepatoblastomas, CNS tumors (especially medulloblastomas), and various benign tumors like adrenal adenomas, osteomas, desmoid tumors, and dental abnormalities (14, 15). In this case, two years and three months after being diagnosed with FAP, a pituitary adenoma was also diagnosed, which could potentially be a new extraintestinal manifestation of FAP.

Familial adenomatous polyposis (FAP) is a rare autosomal dominant disease characterized by germline mutations in the Adenomatous Polyposis Coli (APC) gene (16). APC mutations are also found to be the most frequent mutation in an adult pancreatoblastoma, indicating that APC mutation is a driver mutation of the tumor (6). The APC/β-catenin signalling pathway has also been identified to be associated with 67% of pancreatoblastomas, and abnormalities can include biallelic inactivation of the APC gene and activating mutations of CTNNB1 (β-catenin) gene (7). The above studies suggest that pancreatoblastoma might be associated with FAP by the linkage of APC mutation. A report also shows that there is a non-random association between adult pancreatoblastoma and FAP (17). However, APC mutation was not detected in this case. Therefore, we have no direct proof to confirm the association of pancreatoblastoma and FAP in this only one case.

Pancreatoblastoma often has no obvious clinical symptoms or only presents as abdominal pain, so it is easy to misdiagnose. This patient came to the hospital 1 year and 3 months after being diagnosed with FAP due to abdominal pain induced by incomplete intestinal obstruction caused by FAP, and a pancreatic nodule was incidentally found on abdominal CT, which was first diagnosed as pancreatic neuroendocrine carcinoma with high-differentiation that needed to be differentiated from pancreatoblastoma. Regrettably, this case was not discussed at a multidisciplinary tumor board, as it had not been established in the First Affiliated Hospital of Kunming Medical University until 2020. Therefore, the first diagnosis was not clear and the doctors tended to think of it as pancreatic neuroendocrine carcinoma with high differentiation, according to the Eighth Edition of the AJCC TNM Staging System of Pancreatic Neuroendocrine Tumors, the pathological stage was T2N0M0. The patient was not given chemotherapy after the first pancreatectomy. However, the early recurrence was found seven months after the first pancreatectomy, then the patient underwent the second pancreatic tail resection, and the final diagnosis was pancreatoblastoma. This is a rare case of adult pancreatoblastoma combined with FAP.As the first diagnosis was not accurate, pancreatoblastoma is more malignant than neuroendocrine carcinoma, and some tiny cancerous lesions can’t be identified by the naked eye and imaging examination,the early recurrence was likely due to inadequate resection.

Adult pancreatoblastoma is an exceptionally rare malignant tumor of the pancreas that mimics other solid cellular neoplasms of the pancreas, which may pose diagnostic difficulties. It is important to distinguish pancreatoblastoma from morphological mimics such as pancreatic acinar cell carcinomas, neuroendocrine neoplasms, and solid pseudopapillary neoplasms (5). Pancreatic acinar cell carcinomas are glandular and have amphophilic/eosinophilic cytoplasm, presenting acinar, solid, and trabecular structures (18). Acinar cell carcinomas have a relatively single-cell component, and all tumor cells show the characteristics and immunophenotypes of acinar cells, without squamous corpuscles/squamous epithelial islands. Neuroendocrine neoplasms of the pancreas have different clinical manifestations and laboratory abnormalities depending on the hormones secreted by the tumor, with rich blood sinusoids in the stroma and no squamous corpuscles/squamous corpuscles/squamous epithelial islands (19). Solid pseudopapillary neoplasms in the pancreas often occur in young women and are extremely rare in men. The tumor cells are arranged in the form of solid sheets, micro cysts and pseudopapillary areas that show characteristic pseudopapillae with the fibrovascular axis of the branch-shaped area surrounded by several layers of polygonal epithelioid cells, and there are numerous thin-walled blood vessels or sinuses in the stroma, without squamous corpuscles/squamous epithelial islands. Regional cystic degeneration, haemorrhage, necrosis, aggregates of foamy histiocytes, and cholesterol clefts are common (20, 21).

In this case, the tumor cells in the pancreatic nodule were abundant and separated by fibrous septa into well-defined epithelial cell islands, arranged in large nests. There was acinar cell differentiation and atypical squamous corpuscles/squamous epithelial cell islands, and some cells showed neuroendocrine differentiation. Therefore, according to the characteristic acinar-like cells and squamous corpuscles/squamous epithelial cell islands, this case should be diagnosed as adult pancreatoblastoma. After the second pancreatectomy, five renowned pathological experts from highly regarded tertiary hospitals in China confirmed the final diagnosis as adult pancreatoblastoma.

Adult pancreatoblastomas are aggressive tumours with frequent invasion, recurrence, and distant metastasis (22). Due to the low incidence of pancreatoblastoma, there is a lack of standardized treatment strategies internationally. Surgical resection is the major treatment for pancreatoblastoma. Chemotherapy and radiotherapy may have a role in the treatment of recurrent, residual, unresectable, and metastatic tumors. However, the use of chemotherapy and its efficacy in adult pancreatoblastoma remains unclear, and the prognosis documented in existing literature for adults is worse when compared to paediatric presentations (23).

In this case, the patient received surgical resection of pancreatoblastoma, and pancreatoblastoma recurred in the patient 7 months after the first resection, and the patient received a second resection of the pancreatoblastoma. Abdominal metastases appeared 3 months after the second resection, which is consistent with the clinical characteristics of pancreatoblastoma. Then the patient received 3 cycles of chemotherapy including Vincristine 2mg on Day 1 + Cyclophosphamide 1.0g on Day 2 + Etoposide 0.1g on Day 1 to Day 3 + Cisplatin on Day 3, Day 4, and Day 5. However, this patient was not sensitive to the chosen chemotherapy regimen.

The malignant behaviors of pancreatoblastomas often lead to a poor prognosis. In general, despite aggressive treatment, pancreatoblastoma in adults is associated with poorer outcomes than in children, with a median survival time of 18.5 months (24). In a report of 11 cases of adult pancreatoblastoma, 5(45%) patients died of pancreatoblastoma during the follow-up period(0.8–348 months) (25). In this case, due to poor response to the chosen chemotherapy regimen and intolerance of adverse reactions, the patient discontinued chemotherapy and was discharged. Subsequently, the patient received regular phone follow-ups. Remarkably, the patient has survived for 4 years and 11 months since the diagnosis of pancreatoblastoma till January 2024(last follow-up). However, the treatment details of this patient after discharge were unknown and the imaging data were not obtained.

We also reviewed the data of 18 cases of adult pancreatoblastomas in 6 previous reports (3, 6, 22, 25–27). Age: The average age at diagnosis was 50.4 ± 14.2 years old, with the minimum age being 32 years old and the maximum age being 76 years old. Gender: The male to female ratio is 1:1, suggesting that pancreatoblastomas have no gender predilection. Size: The tumor diameter at diagnosis in all cases was ≥2.5 cm, of which 9 cases (50%) were > 7 cm, and the maximum was 18.7 cm. This suggests that pancreatoblastomas are generally discovered late, which may be caused by asymptomatic or atypical symptoms of PB. Location: Pancreatoblastomas occurred in the pancreatic head in 8 cases, in the pancreatic tail in 8 cases, in the pancreatic body and tail in 1 case and in the pancreatic body in 1 case, suggesting that pancreatoblastomas can occur in any part of the pancreas, but they are more likely to occur in the head and tail of the pancreas. First diagnosis: Among the 18 cases, only 4 cases were initially diagnosed as PB, and 6 cases were initially diagnosed as neuroendocrine neoplasm/carcinoma/tumor. This indicates that the diagnosis of PB is difficult, especially especially when using a small number of biopsy specimens. Neuroendocrine tumors are most similar to PB in morphology and require differential diagnosis from PB. Combined with FAP: There were only 1 cases with FAP and 2 cases with FAP-related syndromes- Gardner syndromes. However, APC mutations were not available in the 3 cases (25). This suggests PB combined with FAP is rare. APC mutations: Only 4 cases were tested for APC mutations, including 2 negative cases and 2 positive cases, but FAP was not found in the 2 positive cases (6, 26). Recurrence:There were only 2 cases of recurrence, indicating that PB is not easy to relapse, and the recurrence of 2 cases might be related to surgical residue. Metastasis:Lymph node or distant metastasis was present in 12 cases(66.7%), and only 6 cases(33.3%) had metastasis, indicating that PB has a high degree of malignancy. Survival period:The longest survival period was >348 months and the shortest survival period was 0.8 months after diadnosis of PB (25). And 13 cases survived for more than 12 months, including 7 cases with a survival period exceeding 60 months. The 1-year survival rate was 72.2%, and the 5-year survival rate was 38.9%, suggesting that some patients with PB have a good prognosis.

Adult pancreatoblastoma can display genetic heterogeneity, diverse histological appearance, and overlapping IHC findings, so the differential diagnosis with other adult pancreatic tumors may be challenging. Although molecular findings might provide useful information, the integration of clinical, radiological, and histopathological findings is essential in pancreatoblastoma diagnosis (26). Here we reported a rare case of adult pancreatoblastoma with atypical histological morphology combined with familial adenomatous polyposis. In this case, we integrated the clinical, radiological, and histopathological findings to make a final diagnosis of pancreatoblastoma. This case report mainly highlights the challenges associated with diagnosing and managing adult pancreatoblastoma.

## 4 Conclusions

This patient was diagnosed with pancreatoblastoma 1 year and 3 months after being diagnosed with FAP. This suggests pancreatoblastoma may occur in patients with familial adenomatous polyposis or a family history of the condition, indicating a possible association between the two tumors. Therefore, pancreatoblastoma should be included in a differential diagnosis for FAP patients with a pancreatic mass. The final diagnosis of pancreatoblastoma depends on the pathological diagnosis. Acinar-like cells and squamous corpuscles/squamous epithelial cell islands under light microscopy are the key diagnostic points. This case report also can improve the awareness of clinicians, radiologists, and pathologists on the presence of rare tumor-adult pancreatoblastoma in patients with familial adenomatous polyposis.

Strengths and limitations of this study Pancreatoblastoma (PB) is a rare malignant epithelial neoplasm of the pancreas that demonstrates multiple patterns of differentiation. In this study, an extremely rare case of adult pancreatoblastoma with atypical histological morphology combined with familial adenomatous polyposis was reported and the clinical, radiological, and morphological features, immunophenotypes, and prognosis of adult pancreatoblastoma combined with familial adenomatous polyposis were summarized. However, APC mutation was not tested in this patient. And this report is only 1 case, our results may need to be verified in more cases.

## Data availability statement

The original contributions presented in the study are included in the article/**Supplementary Material**. Further inquiries can be directed to the corresponding author.

## Ethics statement

The studies involving humans were approved by The Ethical Review Board of the First Affiliated Hospital of Kunming Medical University. The studies were conducted in accordance with the local legislation and institutional requirements. Written informed consent was obtained from the participant/patient(s) for the publication of this case report.

## Author contributions

Y-XW: Data curation, Formal analysis, Investigation, Methodology, Writing – original draft. S-SF: Data curation, Formal analysis, Writing – original draft. X-RP: Data curation, Formal analysis, Writing – original draft. Y-SZ: Data curation, Formal analysis, Writing – original draft. XZ: Conceptualization, Funding acquisition, Supervision, Validation, Writing – review & editing.
