# Supplementary material for: Adult pancreatoblastoma with atypical histological morphology combined with familial adenomatous polyposis: a rare case report
Source: Front Oncol. 2024 Feb 28;14:1346964. doi: 10.3389/fonc.2024.1346964 (PMC10933127; doi:10.3389/fonc.2024.1346964)
Supplement: Supplementary file 1 [file DataSheet_1.pdf]

Table S1 Clinical features of 18 cases of adult pancreatoblastoma

Number	Sex	Age	Tumor size	Location	First diagnosis	Recurrence	Metastasis	Combined with FAP	APC mutaiton	Treatment	Chemotherapy	Survival period	Reference
1	Male	76	31 mm × 20 mm	Head	Malignant epithelial neoplasia compatible with PB	No	No	No	NA	Pyloric-sparing pancreaticoduodenectomy	Yes	>32 months	3
2	Male	74	14.0 ×10.3 ×8.1 cm	Tail	Neuroendocrine tumor	No	Lymph nodes	No	Yes	Distal pancreatectomy	No	>36months	6
3	Female	60	7×7×8 cm	Body and tail	PB	No	Liver	No	NA	Pancreaticoduodenectomy	yes	16 month	22
4	Female	45	80×65×35 mm	Tail	NA	No	Lymph nodes	No	Yes	Distal pancreatectomy	No	> 6 years and 2 months	26
5	Female	39	62×51×32 mm	Tail	NA	No	Liver and lung	No	No	Distal pancreatectomy	Yes	26 months	26
6	Male	69	73×52 mm	Body	NA	No	Liver and peritoneum	No	No	Palliative treatment	No	3 months	26
7	Female	45	18.7 cm	Tail	PB	No	Liver	No	NA	Distal pancreatectomy	Yes	NA	27
8	Male	33	2.8 cm	Head	Neuroendocrine neoplasm	No	No	No	NA	Pancreatectomy	NA	72.2 months	25
9	Male	60	2.5 cm	Head	Neuroendocrine carcinoma	**Yes**	No	No	NA	Pancreatectomy	NA	>17.9 months	25
10	Male	57	5.0 cm	Tail	Positive for malignancy	No	Yes	No	NA	Pancreatectomy	NA	3.6 months	25
11	Male	59	8.7 cm	Head	Neuroendocrine neoplasm	No	Yes	No	NA	Pancreatectomy	NA	85 months	25
12	Female	50	7.5 cm	Head	Acinar cell carcinoma	No	No	Gardner	NA	Pancreatectomy	NA	143.7 months	25
13	Female	43	3.4 cm	Tail	mixed neuroendocrine-acinar cell neoplasma *versus* PB	No	No	Yes	NA	Pancreatectomy	NA	>13.6 months	25
14	Male	34	2.5 cm	Tail	PB	No	Yes	Gardner	NA	Pancreatectomy	NA	0.8 months	25
15	Female	57	10.5 cm	Head	PB	No	No	No	NA	Pancreatectomy	NA	>6.5 months	25
16	Female	34	12.0 cm	Head	Positive for malignancy	No	Yes	No	NA	Pancreatectomy	NA	>348 months	25
17	Male	40	4.0 cm	Head	Neuroendocrine carcinoma	No	Yes	No	NA	Pancreatectomy	NA	>88 months	25
18	Female	32	2.5 cm	Tail	Neuroendocrine tumor	**Yes**	Yes	No	NA	Pancreatectomy	NA	>91 months	25

PB: pancreatoblastoma; FAP: familial adenomatous polyposis; APC: adenomatous polyposis coli；NA: not available.
